# SARS-CoV-2 risk factors among symptomatic vaccinated adults attending community testing locations in the Netherlands from June 2021 till February 2022

**DOI:** 10.1371/journal.pone.0311229

**Published:** 2024-12-30

**Authors:** Claudia Laarman, Susan J. Hahné, Hester E. de Melker, Mirjam J. Knol

**Affiliations:** Centre for Infectious Disease Control, National Institute for Public Health and the Environment (RIVM), Bilthoven, The Netherlands; Universiti Brunei Darussalam, BRUNEI DARUSSALAM

## Abstract

**Introduction:**

Most studies on risk factors for a SARS-CoV-2 infection were conducted in the pre-vaccination era with many non-pharmaceutical prevention measures in place. We investigated risk factors for symptomatic SARS-CoV-2 infections in vaccinated persons in a period with a varying degree of prevention measures.

**Methods:**

In a test-negative case control study among vaccinated adults attending community COVID-19 testing locations between June 1^st^ 2021 till February 28^th^ 2022, we compared symptomatic cases with symptomatic controls (to study risk factors specific for SARS-CoV-2) and with asymptomatic controls (to study risk factors that could apply to respiratory infections in general). We examined potential risk factors including household composition and mitigation behaviour by logistic regression, adjusting for age, sex, and week of testing.

**Results:**

Risk factors for a positive SARS-CoV-2 test when symptomatic cases were compared to symptomatic controls were: having a household size of more than 4 (adjusted odds ratio: 1.47; 95% CI 1.14–1.92), being a healthcare worker (1.27;1.18–1.47), and visiting busy locations outside (1.49;1.19–1.87). When symptomatic cases were compared to asymptomatic controls, a household size of more than 4 members (1.71;1.25–2.33), living with children aged 0–12 (1.59;1.12–2.26), visiting busy locations outside (1.64;1.24–2.17) were independent risk factors for a positive SARS-CoV-2 test. Risk factors for separate periods and waves differed from the study period as a whole.

**Conclusion:**

This study was conducted in a period with a varying degree of prevention measures. Among vaccinated individuals, we identified several SARS-CoV-2 specific risk factors and SARS-CoV-2 risk factors that could be more general for respiratory infections. For SARS-CoV-2 transmission more attention could be given to visiting busy outdoor locations, having a household size that consists of more than 4 persons, being a healthcare worker, and living with children aged 0–12. Risk factors varied with different phases in the pandemic, emphasizing the importance of repeated assessment of risk factors.

## Introduction

From the beginning of the COVID-19 pandemic, the Netherlands have had twelve waves of hospital admissions up until the summer of 2023 [1]. In 2021 solely, the COVID-19 pandemic has led to an estimated burden of disease with a total of 218,900 (215,300–222,600) DALYs lost in the Netherlands. In 2021, 19.4 thousand inhabitants died due to COVID-19 in a population of approximately 17.5 million inhabitants [2]. Multiple restrictions and preventive interventions were taken by the government to slow down the spread of the SARS-CoV-2 virus up and until March 2022 [3]. On January 6^th^, 2021 the Netherlands started its COVID-19 vaccination campaign [4].

Insight into risk factors for a SARS-CoV-2 infection is important to inform prevention and control measures. Most studies on risk factors for a SARS-CoV-2 infection were conducted in the pre-vaccination era in the context of many non-pharmaceutical prevention measures. From a public health perspective it is important to keep investigating risk factors for a SARS-CoV-2 infection, because of the changing nature of the SARS-CoV-2 virus and the interventions to control it. SARS-CoV-2 still causes considerable morbidity and mortality [5] and can lead to post-COVID-19 condition [6]. Thereby, lessons can be learnt for future epidemics and pandemics of respiratory pathogens. Risk factors found in the beginning of the pandemic were for example having contact with a known SARS-CoV-2 case, going to work, using public transport, attending crowded spaces, having activities outside of home that not included grocery shopping or visiting the pharmacist and the number of non-household contacts [7–14]. However, risk factors for the period when people could be vaccinated for COVID-19 and when other variants of SARS-CoV-2 were circulating are mainly unknown. Risk factors for a SARS-CoV-2 infection in the vaccination-era are of particular interest, since people may alter their behaviour regarding prevention measures against SARS-CoV-2 when they are vaccinated [15]. Knowledge about SARS-CoV-2 risk factors in a period with vaccine coverage is valuable, because of post-COVID-19 condition and mortality, but also because hospital admissions could increase with new emerging variants that could lead to a worse manifestation of COVID-19.

Thereby it is interesting to know if risk factors differ between certain periods of time in a year when another variant is dominant. For instance, a Japanese study suggested that certain risk factors, e.g. spending ≥ 30 minutes in closed spaces and crowded spaces, were risk factors for infection with the Omicron variant but not the Delta variant [16]. This may result in other risk factors for October 2021 till December 2021 than from January and February 2022. Making a further distinction in periods could help prevention and control.

We conducted a test-negative case-control study to investigate risk factors for a positive SARS-CoV-2 test among vaccinated persons who got tested at the PHS testing facilities in the Netherlands between June 1^st^ 2021 until February 28^th^ 2022. In this period everyone in the Netherlands was eligible for COVID-19 vaccination. At the start of this period more than 70% of all people above the age of 55 years had at least received one COVID-19 vaccine. At the end of December 2021, approximately 75% of all people above the age of 18 years had received at least one COVID-19 vaccine {, 2021 #22}{, 2021 #23}. We included symptomatic cases and both symptomatic controls and asymptomatic controls. By doing so, we could determine specific risk factors for a SARS-CoV-2 infection in individuals with symptoms as well as SARS-CoV-2 risk factors that could also be more general to other respiratory infections. Moreover, we repeated the analyses for certain periods and waves in the period June 1^st^ 2021 up and till February 28^th^ 2022 to investigate if there were differences in risk factors for a positive SARS-CoV-2 test.

## Methods

### Study design

For this study we used data from the CONTEST study, which was a test-negative case-control study among adults attending community SARS-CoV-2 testing facilities in the Netherlands from February 8 2021 till March 11 2022. These free-of-charge SARS-CoV-2 testing facilities were set up by the Dutch Public Health Services (PHS). People could access the testing facilities by foot, bike or car throughout the Netherlands. Appointments for a SARS-CoV-2 test could be made online or by telephone. Different kind of tests were used during the study period, including a reverse transcriptase polymerase chain reaction (RT-PCR), a rapid antigen test, and a loop-mediated isothermal amplification (LAMP). Persons could be tested at the PHS when they experienced COVID-19-like symptoms, had contact with a person that was tested positive for SARS-CoV-2, when they returned from a country that was assigned as high-risk by the Dutch government, for confirmation of a self-administered rapid antigen test, or if they received a notification through the CoronaMelder application. The CoronaMelder application sent an alert to all persons whose mobile phone was in close proximity for at least 15 minutes with a person who was tested positive for SARS-CoV-2. During the study period, people could not get tested at the PHS for a certificate for travelling or attending events.

Persons who made an appointment for a SARS-CoV-2 test at the testing facility were invited to participate in our study via a link in the confirmation email of the test appointment. Persons were eligible to participate when aged ≥ 18 years old, previously not participating in the study, completing the questionnaire before receiving the test result, and not residing at a care facility.

This study was reviewed by the Centre for Clinical Expertise (KEC) (EPI-456) at the RIVM and considered not being subject to the Act on Research Involving Human Subjects (WMO). Participation in the questionnaire was voluntary and online informed consent was obtained by ticking a box before filling out the questionnaire.

### Data collection

We used an online questionnaire to collect information on sociodemographics (e.g. age, sex, education level, household size), vaccination history, symptoms, comorbidities, and exposure-related variables in the previous 14 days such as mask wearing, and number of contacts at work or study. Persons were asked to fill in the questionnaire before they had their test appointment.

Persons received a unique identifier when they made an appointment for testing at the PHS testing facility. Participants were asked to fill in this unique identifier in the questionnaire, through which test results were linked to the questionnaire data.

### Study population

In the current analysis, we only included participants who were tested between June 1^st^ 2021 and February 28^th^ 2022 and were fully vaccinated. Persons were defined as fully vaccinated if they received their second dose of Comirnaty, Spikevax or Vaxzevria ≥14 days before onset of symptoms for symptomatic participants or ≥14 days prior to getting tested for asymptomatic participants. An interval of ≥28 days was used for participants that were vaccinated with a single dose of Jcovden (Janssen).

We excluded participants that were partially vaccinated, received three doses of vaccine, had a household member that was tested positive for SARS-CoV-2, and reported a positive self-administered antigen test as reason for testing (to make sure that included participants were blinded to their test results when filling out the questionnaire). The reasoning behind these exclusion criteria were that we aimed to investigate the risk factors for a positive SARS-CoV-2 test in individuals who were considered as fully vaccinated without booster vaccination in a homogeneous group. Additionally, we wanted to study symptomatic individuals who were infected with SARS-CoV-2 in the community and not in their own household. We stratified the participants into three groups, namely symptomatic cases, symptomatic controls, and asymptomatic controls. Asymptomatic cases were excluded from the analysis. Participants were divided into these groups to study risk factors specific for SARS-CoV-2 (symptomatic cases vs. symptomatic controls) and risk factors for SARS-CoV-2 which may be more general for a respiratory infection (symptomatic cases vs. asymptomatic controls) [17].

### Risk factors

The following risk factors were included in the analysis: total household size, living with children aged 0–12, living with children aged 13–18, being a healthcare worker, went to work or study, close contact at work or school, visiting a busy indoor location, visiting a busy outdoor location, mask wearing inside public spaces, mask wearing in outside public spaces. All variables focused on the 14 days before completing the questionnaire. Busy was defined as not being able to keep a 1.5 meter distance.

Total household size was categorized into 1, 2 to 3 and ≥ 4 persons. A participant was considered as a healthcare worker when the person was working in the healthcare sector and had patient contact. Patient contact could be on more or less than 1.5 meter distance.

The variable “went to work/school” encompasses going to work or going to school in the previous 14 days and was categorized into went to work or school for 1 to 5 days, 6 to 10 days, or more than 10 days, did not go to work/school, and does not have to go to work/school. The category ‘does not have to go to work’ encompasses persons that could work fully remote as well as people that do not have to work, e.g. retired persons. Close contact (defined as less than 1.5 meter distance) at work or school in the previous 14 days was categorized into 0, 1–4, 5–9, 10–19, and 20+ contacts.

Mask wearing behaviour was categorized into two categories: always/mostly and sometimes/rarely/never.

### Data analysis

Logistic regression models with symptomatic cases and either symptomatic or asymptomatic controls were used to determine risk factors for SARS-CoV-2 infections. The effect of each risk factor was assessed in a separate model, adjusted for age groups (18–29, 30–44, 45–59, 60–69, 70+), gender, and week of testing. Week of testing was analyzed with penalized splines to correct for differences in time e.g. restrictions and responses that were taken by the government. Each model was built with test result as dependent variable, risk factor as independent variable, and age, sex, and week of testing with penalized splines as covariates. Two sensitivity analyses were performed. One sensitivity analysis was conducted with additional adjustment for days since vaccination. In another sensitivity analysis the logistic regression models were also conducted in participants that received at least one vaccine.

Analyses were done for the whole study period and stratified by periods and infection waves. The COVID-19 pandemic in the Netherlands was divided into six distinct periods based on the number of infection notifications, hospital admissions, restrictive measures, and circulating and emerging variants [18]. Our study period encompasses the fourth period (July 4^th^, 2021 up and till October 3^rd^, 2021) by using data from June 1^st^ 2021 up and till October 3^rd^, 2021 and coincided with the fifth period (October 4^th^ 2021 and ended on January 2^nd^, 2022) Each period is characterized by a peak in virus circulation, hospital admissions, and (general) mortality. Period 4 differs from period 5 in level of restrictions (being fewer in most of period 4 compared to period 5) and vaccine coverage was higher in period 5. In both waves the Delta variant was the dominant variant, however in the last week of period 5 Omicron BA.1 was the dominant variant. For the analyses, period 4 and 5 were named period A and period B, respectively. In addition, two specific infection waves occurred during our study period: one in the summer of 2021 (28-6-2021 up and till 2-08-2021) and another in the fall of 2021 (4-10-2021 up and till 20-12-2021). The total number of notifications of positive SARS-CoV-2 tests were used to determine both infection waves. These waves are referred to as the summerwave and the fallwave.

Statistical analyses were conducted with R versions R 4.2.1 and R 4.2.2.

## Results

### Study population

**Fig 1:**
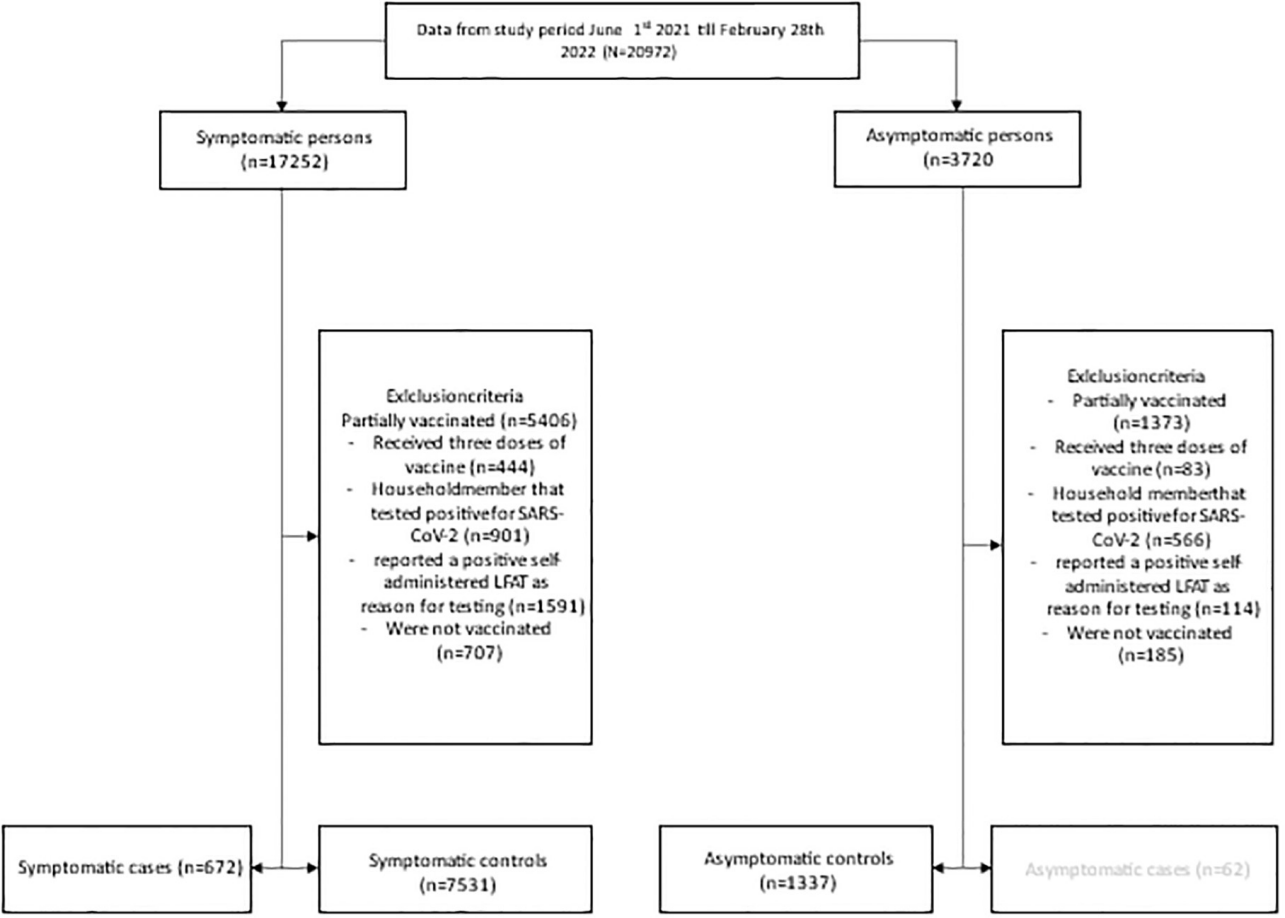
Flowchart of the exclusion criteria of symptomatic cases, symptomatic controls and asymptomatic controls of the CONTEST study in the period June 1^st^ 2021 till February 28^th^ 2022.

From June 1st 2021 till February 28^th^ 2022, 20972 persons participated in the study by filling in the questionnaire. Among 17252 participants with symptoms at time of testing, we excluded 9049 participants because of being partially vaccinated, receiving three doses of vaccine, having a household member that was tested positive for SARS-CoV-2, reporting a positive self-administered test, and not being vaccinated (Fig 1). This resulted in a total number of 8203 symptomatic participants of whom 672 tested positive for SARS-CoV-2 and 7531 tested negative. For the 3720 participants who had no symptoms at time of testing the same exclusion criteria were applied, which resulted in 1399 asymptomatic participants of whom 1337 tested negative for SARS-CoV-2 and were included as asymptomatic controls.

Of the included participants (n = 9540), the median ± IQR age was 51 ± 29 years old. The age groups had the following distribution: 18 to 29 (n = 1809, 19%), 30–44 (n = 2014, 21%), 45–59 (n = 2810, 29%), 60–69 (n = 2021, 21%), and 70 years old or more (n = 886, 9%). Of the participants with known gender 67% was female (n = 6396) and 33% was male (n = 3118). Two-third of participants had a higher education level (67%, n = 6402) and 2% (n = 204) were lower educated. In addition, 92% (n = 8788) was born in the Netherlands.

### Overall study period

All tables and figures of the sensitivity analyses can be found in the supplemental material, as well as an overview of all statistically significant risk factors (S1 Table in S1 File).

#### Symptomatic cases versus symptomatic controls

**Fig 2:**
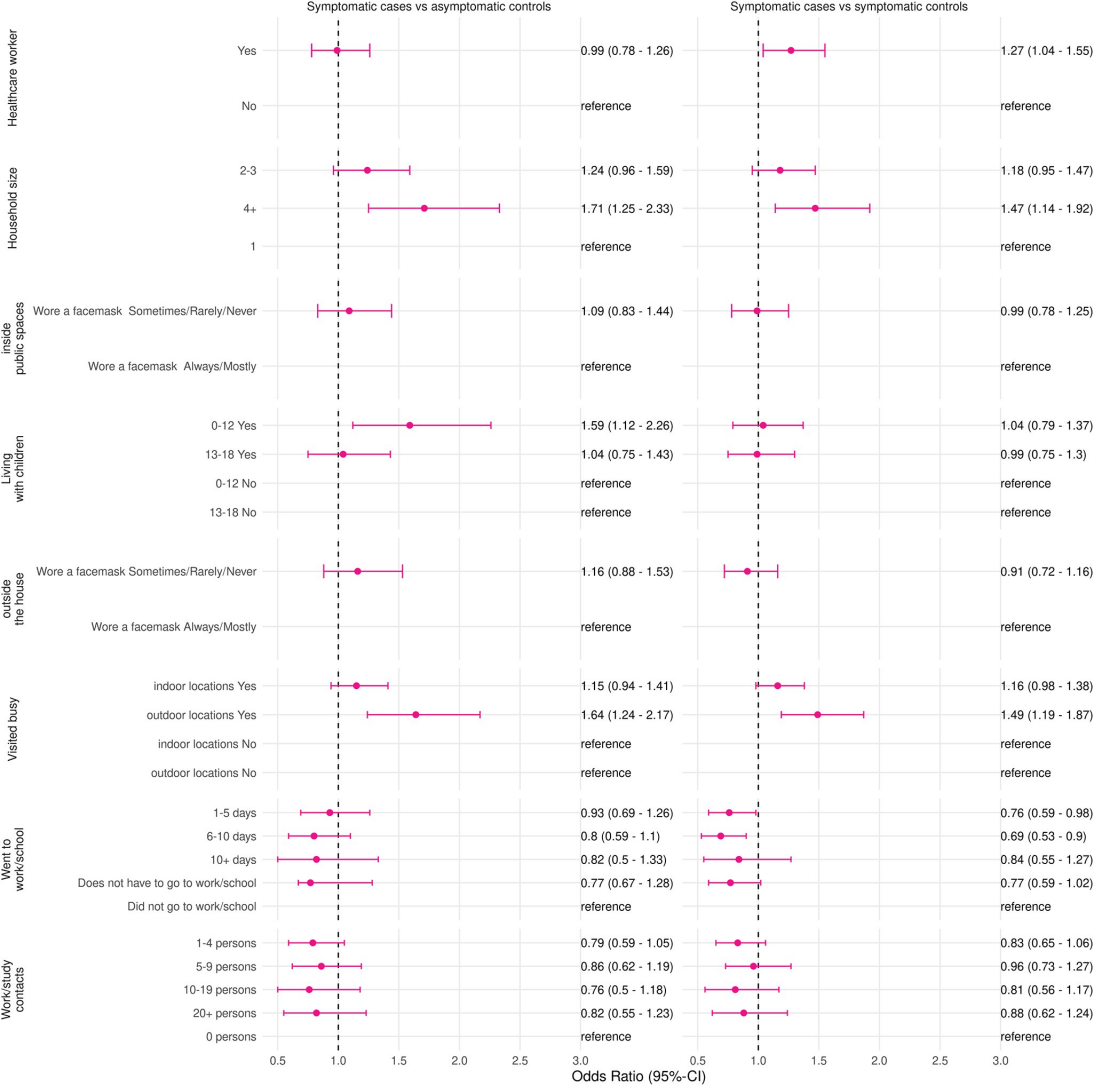
Adjusted odds ratios and 95%-confidence intervals of risk factors for a positive SARS-CoV-2 test in fully vaccinated persons, comparison of symptomatic cases with symptomatic controls and asymptomatic controls in the Netherlands, 1 June 2021–28 February 2022. Adjusted for age group, gender, and week of testing.

Risk factors for a positive SARS-CoV-2 test when symptomatic cases were compared to symptomatic controls were: having a household size of more than 4 members (adjusted OR 1.47; 95% CI 1.14–1.92), working as a healthcare worker (adjusted OR 1.27;1.18–1.47), and visiting busy outdoor locations (adjusted OR 1.49;1.19–1.87) (Fig 2 and S2 Table in S1 File). Went to work for 1–5 days (adjusted OR 0.76;0.59–0.98) and 6–10 days (adjusted OR 0.69;0.53–0.90) were protective factors for a positive SARS-CoV-2 test.

*Sensitivity analyses*. In sensitivity analysis for participants who had received at least one COVID-19 vaccine it was shown that having a household size of more than 4 (1.30;1.06–1.60), visiting busy outdoor locations (1.37;1.13–1.66) and busy indoor locations were statistically significant risk factors (S1 Fig and S4 Table in S1 File). When we additionally adjusted for days since vaccination the same risk factors were found as in the main analyses (S6 Table and S2 Fig in S1 File).

#### Symptomatic cases versus asymptomatic controls

Risk factors for a positive SARS-CoV-2 test when symptomatic cases were compared to asymptomatic controls were: a household size of more than 4 members (adjusted OR 1.71;1.25–2.33), living with children aged 0–12 (adjusted OR 1.59;1.12–2.26), and visiting busy outdoor locations (adjusted OR 1.64;1.24–2.17) (Fig 2 and S3 Table in S1 File).

*Sensitivity analyses*. In the sensitivity analyses the same risk factors were found as in the main analysis (S5, S7 Tables and S1, S2 Figs in S1 File). With the exception of living with children aged 0–12 which was not statistically significant for participants that had at least one vaccine.

### Periods and waves

#### Symptomatic cases and symptomatic controls

**Fig 3:**
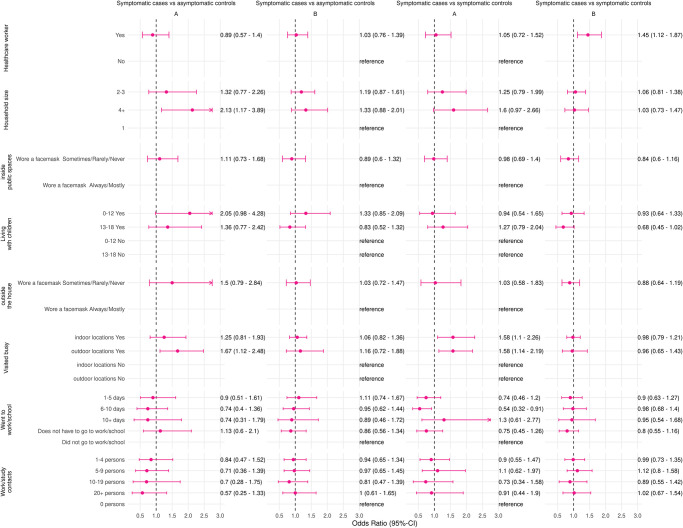
Adjusted odds ratios and 95%-confidence intervals of risk factors for a positive SARS-CoV-2 test in fully vaccinated persons, comparison of symptomatic cases with symptomatic controls and asymptomatic controls in the Netherlands, for the periods 1 June 2021–3 October 2021 (period A) and 4 October 2021–2 January 2022 (period B). Adjusted for age group, gender, and week of testing. An arrow indicates that the upper 95%-confidence limit is not fully shown in the figure.

**Fig 4:**
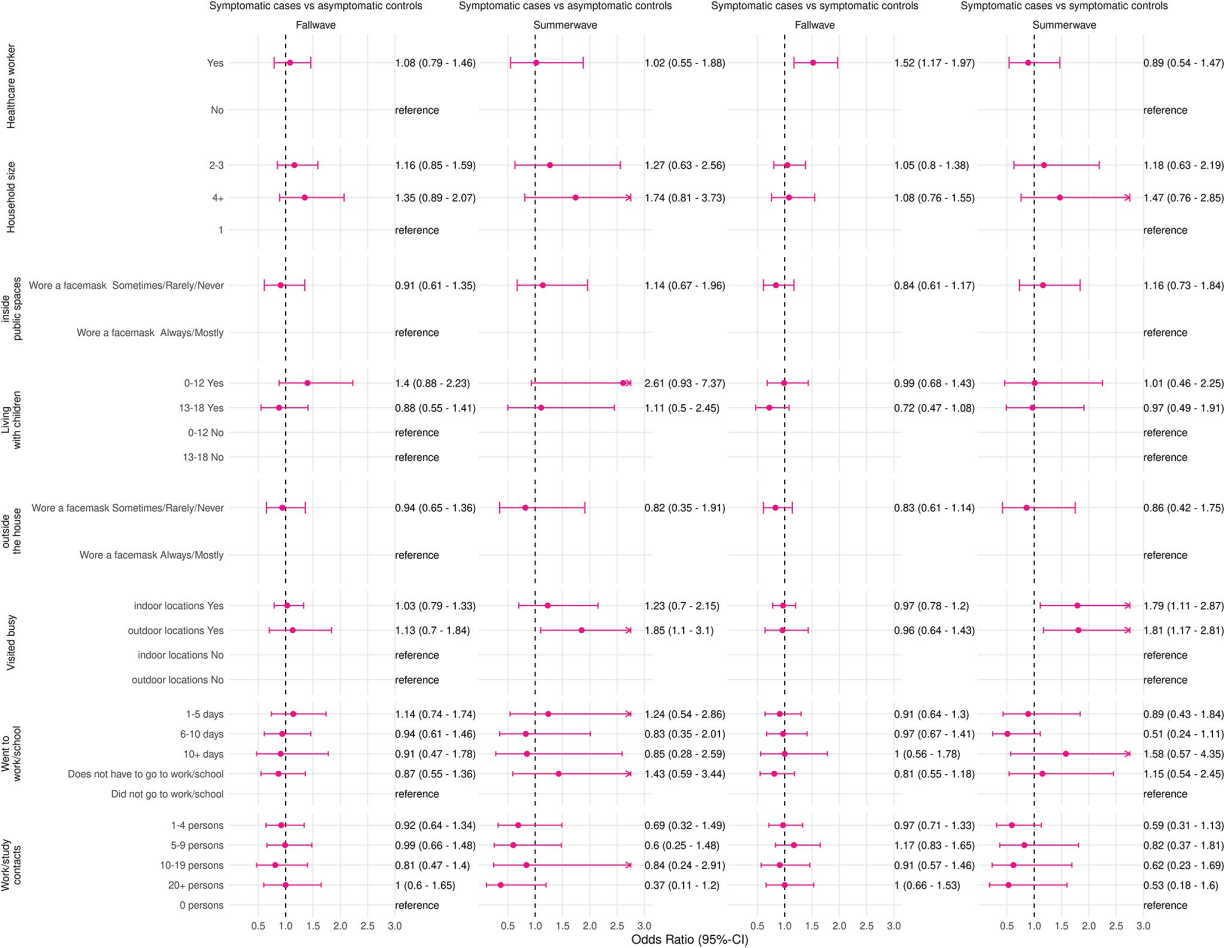
Adjusted odds ratios and 95%-confidence intervals of risk factors for a positive SARS-CoV-2 test in fully vaccinated persons, comparison of symptomatic cases with symptomatic controls and asymptomatic controls in the Netherlands, for the summerwave of 2021 (28 June 2021–2 August 2021) and the fallwave of 2021 (4 October 2021–20 December 2021). Adjusted for age group, gender, and week of testing. An arrow indicates that the upper 95%-confidence limit is not fully shown in the figure.

In period A when there were less restrictions in the Netherlands and vaccine coverage was lower compared to the overall study period the risk factors visiting busy indoor locations (adjusted OR 1.58; 1.10–2.26) and visiting busy outdoor locations (adjusted OR 1.58;1.14–2.19) and went to work for 6–10 days (adjusted OR 0.55;0.33–0.92) were statistically significant risk factors (Fig 3 and S8 Table in S1 File). During the summer wave of 2021 there were two risk factors: visiting busy indoor locations (adjusted OR 1.79;1.11–2.87) and visiting busy outdoor locations (adjusted OR 1.81;1.17–2.81; Fig 4 and S12 Table in S1 File).

In period B more restrictions were in place than during period A or the whole study period and vaccine coverage was higher. In this period being a healthcare worker (adjusted OR 1.45; 1.12–1.87) was the only risk factor found (Fig 3 and S9 Table in S1 File). Moreover, being a healthcare worker was the only risk factor for the fallwave of 2021 (adjusted OR 1.45;1.12–1.87; Fig 4 and S13 Table in S1 File).

#### Symptomatic cases and asymptomatic controls

Two risk factors for a positive SARS-CoV-2 test in period A were found when there were less restrictions in place and vaccine coverage was lower compared to three risk factors for the whole study period (Fig 3 and S10 Table in S1 File). These risk factors were having a household size of more than 4 (adjusted OR 2.13;1.17–3.89) and visiting busy outdoor locations (adjusted OR 1.67;1.12–2.48). One risk factor was identified for the summer wave of 2021 (Fig 4 and S14 Table in S1 File). This risk factor was visiting busy outdoor locations (adjusted OR 1.85;1.10–3.10).

For both period B and the fallwave of 2021 no statistically significant risk factors for a positive SARS-CoV-2 test were found (Figs 3 and 4 and S11 and S15 Tables in S1 File). In this period and wave there were more restrictions in place and vaccine coverage was higher compared to period A, the summerwave of 2021, and the whole study period.

## Discussion

We investigated risk factors for a SARS-CoV-2 test in vaccinated adults in the Netherlands in a period when the entire population had had the opportunity to be vaccinated and when there were still several physical distancing measures implemented, albeit at a varying degree (June 1^st^ 2021 until February 28^th^ 2022). For this study we used both symptomatic controls to investigate specific risk factors for a SARS-CoV-2 infection and asymptomatic controls to study SARS-CoV-2 risk factors that could also be more general for respiratory infections. We found that a household that consisted of 4 or more persons and visiting busy outdoor locations were risk factors for a symptomatic SARS-CoV-2 infection, both when using symptomatic and asymptomatic controls. Living with children aged 0–12 was a risk factor when using asymptomatic controls. Working as a healthcare worker was a risk factor for SARS-CoV-2 among symptomatic participants, while going to work or study prior to getting symptomatic was a protective factor for SARS-CoV-2 among symptomatic participants. No statistically significant associations were found for the amount of contacts at work or study, living with children aged 13–18, and usage of facemasks.

We found that visiting busy outdoor locations was a risk factor for a symptomatic SARS-CoV-2 infection when both symptomatic and asymptomatic controls were used. This is contradictory to a systematic review which found that SARS-CoV-2 transmission and transmission of other respiratory viruses is higher in indoor settings compared to outdoor settings [19]. The measures that were in place in indoor environments could be an explanation for our surprising finding. Indoor spaces were adapted to keep 1.5 meter distance of each other, facemasks were mandatory, and from September 2021 till February 2022 people needed to prove they were recovered from COVID-19, were tested negative or were vaccinated to enter a variety of public indoor spaces, e.g. the gym and restaurants. All these measures could have led to less transmission of the SARS-CoV-2 virus in indoor spaces. In addition, we do not know the kind of activities that were outside, the density of the people at the outdoor location, the duration of the outside activity, and whether or not protective measures were used. This all could have an influence on the result that visiting a busy outdoor location was found as a risk factor.

Strikingly, we found that going to work or study prior to getting symptoms was protective for a positive SARS-CoV-2 test. In this period people had to go in quarantine when they had contact with a person that was tested positive for SARS-CoV-2 to prevent transmission to other people. These were rules at work places and at schools. People that went to work or study possibly did not have had contact with a known case and people that were tested positive for SARS-CoV-2 or had contact with a case were not allowed to visit their workplace or school. Moreover, most people were allowed to go to their workplace or school when they were tested negative for SARS-CoV-2, but were symptomatic probably due to a respiratory virus other than SARS-CoV-2. Perhaps this could be a reason why we found that going to work/study was only a protective factor for a positive SARS-CoV-2 test in symptomatic people.

We found that having children aged 0–12 was a risk factor for symptomatic SARS-CoV-2, but only in the analysis using asymptomatic controls. A longitudinal study that investigated the circulation of respiratory viruses in households with children from 2010 to 2013 found that most respiratory infections were seen in children aged 0–4 and in adults that were in contact with this particular age group [20]. Another longitudinal study about endemic coronaviruses that was conducted before the COVID-19 pandemic found that the highest infection frequency of endemic coronaviruses was seen in children aged 0–4 years [21]. Mostly young children are vulnerable for various kinds of respiratory infections. This may be an explanation why we found that living with children aged 0–12 was only a risk factor for SARS-CoV-2 when symptomatic cases were compared to asymptomatic controls. A systematic review and two meta-analyses that investigated the household transmission of SARS-CoV-2 did not find a dominant role for children in this transmission [22, 23]. However, the systematic review and meta-analyses stated that the transmissibility of COVID-19 was increasing with new emerging variants of SARS-CoV-2, though not statistically significant [22]. The abovementioned information and our results indicate that it is likely that SARS-CoV-2 could transmit from child to adult like other respiratory infections (including endemic coronaviruses).

We did not find an association between facemask usage and a positive SARS-CoV-2 test. No consensus is reached on the effect of wearing face masks on reducing transmission of SARS-CoV-2 and other respiratory infections in community settings. Most studies investigating the use of facemasks are conducted in the household context or in specific settings, e.g. pilgrimage [24–26]. Studying the effect of wearing facemasks on respiratory infection incidence remains difficult. One of the reasons entails the difficulties of studying behaviour. A lot of components can influence a persons’ behaviour in facemask use, e.g. intention and attitudes. For SARS-CoV-2, risk perceptions on chance of infection and fatality may play a role in wearing facemasks if they are not obligatory to wear [27]. The data we had on the use of facemasks may have been inadequate to study their effect. First, using, reusing, and taking a facemask off all need to happen in a specific way [28]. Suboptimal use of facemasks is unlikely to contribute to infection prevention. Second, also the type of the facemask may be of importance. Many types of facemasks exists. There was no specific advice on what type of facemask to use in the Netherlands till the Omicron variant emerged [29]. Many studies do not investigate the use of cloth/fabric masks [25], which was one of the types used in the Netherlands.

For the comparison between symptomatic cases and asymptomatic controls no risk factors for a positive SARS-CoV-2 test were found in the period October 4^th^ 2021-January 2^nd^ 2022 and in the fallwave. In the comparison between symptomatic cases and symptomatic controls only the factor being a healthcare worker was statistically significant. On the contrary, for the period June 1^st^-October 3^rd^ 2021 and the summerwave multiple risk factors were found when symptomatic cases were compared to both symptomatic controls and asymptomatic controls. In the period October 4^th^ 2021-January 2^nd^ 2022 and the fallwave more preventive measures were taken by the government, and eventually a lockdown was installed at end of December 2021. This could be a reason why no risk factors for COVID-19 were found in our study for the period October 4^th^ 2021-January 2^nd^ 2022 and the fallwave of 2021. It is likely that people were infected by other risk factors that we did not measure and investigate.

While visiting a busy indoor location was found to be a risk factor among symptomatic persons in the period June 1^st^-October 3^rd^ 2021 and the summerwave of 2021, this was not a risk factor for the study period as a whole. This difference might be explained by the same reason as mentioned above about the differences in periods.

### Strengths

In this study, we were able to use two different control groups. Including both symptomatic and asymptomatic controls in a case-control study increases the usefulness of the study, because both specific risk factors as well as risk factors that may be general for respiratory infections could be studied. Also, we used controls that got tested at the PHS instead of random population controls. By using controls that went testing, health care seeking behaviour will be the same for both groups. When there is a difference in the factors on which the decision to go to the PHS for a SARS-CoV-2 test is based between cases and controls, this could lead to weaker associations. Furthermore, recall bias was minimized in this study as participants were recruited and filled in their questionnaire before they knew their test results.

### Limitations

A limitation of our study is that the study population may not be representative for the whole Dutch population. Mainly the age groups 45–59 and 60–69 are overrepresented in this population for analyses compared to the groups 18–29 and 70+ [30]. Furthermore, more people were born in the Netherlands in the population for analyses compared to the general population as well as the source population [31]. This could be a threat to the external validity of the study. Results cannot be extrapolated to the whole Dutch population or to populations of other countries.

In addition, it is likely that our study population was relatively health conscious, since they were invited among people who attended a PHS for testing. These people may live and behave more cautiously than those who do not attend a PHS test service. Because of this, not all risk factors may be found or odds ratios may be underestimated.

Moreover, by using a test-negative design with both symptomatic and asymptomatic controls the assumption is made that symptomatic controls are infected by a pathogen other than SARS-CoV-2 and the asymptomatic controls are not infected by a pathogen at all. In the summer months fewer respiratory viruses are circulating in the population. People can also have symptoms due to non-infectious reasons, e.g. sore throat due to stress or nasal cold due to allergies. Because of this, it may be that risk factors that were found in the comparison between symptomatic cases and symptomatic controls do not always reflect specific risk factors for SARS-CoV-2, because symptomatic controls may experience symptoms due to non-infectious reasons. In future research, it is important to study the winter months when more respiratory viruses are circulating and only take samples of participants with a known respiratory infection for the symptomatic control group.

Finally, some answer categories had to be merged to make sure that we had enough power for the analyses. For example it would be interesting to know the odds for a positive SARS-CoV-2 test for contact professions and a distinction in several types of healthcare workers. A Danish cohort study found for example that risk of a SARS-CoV-2 infection differed between different healthcare professions, e.g. nurses and physiotherapists [32]. By making the categories more narrow, results may be more specific which could help in making public health advice and policy.

### Conclusion

This study was conducted in a period of time with a varying degree of prevention measures. Among vaccinated individuals, we identified several risk factors for symptomatic SARS-CoV-2, that differed between various periods (with different preventive measures) as well as between symptomatic (SARS-CoV-2 specific risk factors) and asymptomatic controls (both SARS-CoV-2 and general respiratory infection risk factors). This study shows the complexity to gain adequate insight into relevant risk factors and shows that risk factors need to be interpreted in the context of the preventive measures in place and the dominant variant that is circulating in the population.

## Supporting information

S1 File S1-S15 Tables and S1 and S2 Figs. (DOCX)
