# Supplementary material for: SARS-CoV-2 risk factors among symptomatic vaccinated adults attending community testing locations in the Netherlands from June 2021 till February 2022
Source: PLoS One. 2024 Dec 30;19(12):e0311229. doi: 10.1371/journal.pone.0311229 (PMC11684634; doi:10.1371/journal.pone.0311229)
Supplement: S1 File — (DOCX) [file pone.0311229.s001.docx]

Supplemental material – Tables S1-S15 and Figures S1 and S2

**Table S1: Overview of statistically significant risk factors for the whole study period, Periods A and B, summerwave and fallwave. A pink arrow (**↑**)** **indicates symptomatic cases versus symptomatic controls. A purple arrow (**↑**)** **indicates symptomatic cases versus asymptomatic controls. An upward arrow (↑) indicates a statistically significant risk factor. An downward arrow (**↓**)** **indicates a statistically significant protective factor.**

| **Risk factor** | **Whole Study period** | **Period A** | **Period B** | **Summerwave** | **Fallwave** |
| --- | --- | --- | --- | --- | --- |
|  | **1 June 2021- 28 February 2022** | **1 June 2021 – 3 October 2021** | **4 October 2021 - 2 January 2022** | **28 June 2021 – 2 August 2021** | **4 October 2021 – 20 December 2021** |
| Total household size |  |  |  |  |  |
| 1 (ref) |  |  |  |  |  |
| 2-3 |  |  |  |  |  |
| 4+ | ↑↑ | ↑ |  |  |  |
| Living with children 0-12 |  |  |  |  |  |
| No (ref) |  |  |  |  |  |
| Yes | ↑ |  |  |  |  |
| Living with children 13-18 |  |  |  |  |  |
| No (ref) |  |  |  |  |  |
| Yes |  |  |  |  |  |
| Healthcare worker |  |  |  |  |  |
| No (ref) |  |  |  |  |  |
| Yes | ↑ |  | ↑ |  | ↑ |
| Days went to work/study |  |  |  |  |  |
| Did not go to work/study (ref) |  |  |  |  |  |
| 1-5 days | ↓ |  |  |  |  |
| 6-10 days | ↓ | ↓ |  |  |  |
| 10+ days |  |  |  |  |  |
| Does not have to go to work/study |  |  |  |  |  |
| Contacts at work/study |  |  |  |  |  |
| 0 contacts (ref) |  |  |  |  |  |
| 1-4 contacts |  |  |  |  |  |
| 5-9 contacts |  |  |  |  |  |
| 10-19 contacts |  |  |  |  |  |
| 20 or more contacts |  |  |  |  |  |
| Visited busy indoor locations |  |  |  |  |  |
| No (ref) |  |  |  |  |  |
| Yes |  | ↑ |  | ↑ |  |
| Visited busy outdoor locations |  |  |  |  |  |
| No (ref) |  |  |  |  |  |
| Yes | ↑↑ | ↑↑ |  | ↑↑ |  |
| Wore a facemask outside the house |  |  |  |  |  |
| Always/Mostly (ref) |  |  |  |  |  |
| Sometimes/Rarely/Never |  |  |  |  |  |
| Wore a facemask inside public spaces |  |  |  |  |  |
| Always/Mostly (ref) |  |  |  |  |  |
| Sometimes/Rarely/Never |  |  |  |  |  |

**Table S2: Adjusted* odds ratios and 95%-confidence intervals of risk factors for a positive SARS-CoV-2 test in fully vaccinated persons, comparison of symptomatic cases with symptomatic controls in the Netherlands, 1 June 2021 – 28 February 2022. Adjusted for age group, gender, and week of testing.**

| **Risk factor** | **Cases (n)** | **Controls (n)** | **OR** | **95%-CI** |
| --- | --- | --- | --- | --- |
| Total household size |  |  |  |  |
| 1 (ref) | 118 | 1552 |  |  |
| 2-3 | 388 | 4389 | 1.18 | 0.95-1.47 |
| 4+ | 166 | 1620 | 1.47 | 1.14-1.92 |
| Living with children 0-12 |  |  |  |  |
| No (ref) | 582 | 6446 |  |  |
| Yes | 90 | 1085 | 1.04 | 0.79-1.37 |
| Living with children 13-18 |  |  |  |  |
| No (ref) | 601 | 6599 |  |  |
| Yes | 71 | 932 | 0.99 | 0.75-1.30 |
| Healthcare worker |  |  |  |  |
| No (ref) | 517 | 5898 |  |  |
| Yes | 155 | 1633 | 1.27 | 1.18-1.47 |
| Days went to work/study |  |  |  |  |
| Did not go to work/study (ref) | 115 | 951 |  |  |
| 1-5 days | 195 | 2394 | 0.76 | 0.59-0.98 |
| 6-10 days | 166 | 2221 | 0.69 | 0.53-0.90 |
| 10+ days | 35 | 348 | 0.84 | 0.55-1.27 |
| Does not have to go to work/study | 161 | 1617 | 0.77 | 0.59-1.02 |
| Contacts at work/study |  |  |  |  |
| 0 contacts (ref) | 439 | 4492 |  |  |
| 1-4 contacts | 86 | 1169 | 0.83 | 0.65-1.06 |
| 5-9 contacts | 70 | 816 | 0.96 | 0.73-1.27 |
| 10-19 contacts | 36 | 495 | 0.81 | 0.56-1.17 |
| 20 or more contacts | 41 | 559 | 0.88 | 0.62-1.24 |
| Visited busy indoor locations |  |  |  |  |
| No (ref) | 378 | 4299 |  |  |
| Yes | 294 | 3232 | 1.16 | 0.98-1.38 |
| Visited busy outdoor locations |  |  |  |  |
| No (ref) | 548 | 6438 |  |  |
| Yes | 124 | 1093 | 1.49 | 1.19-1.87 |
| Wore a facemask outside the house |  |  |  |  |
| Always/Mostly (ref) | 97 | 775 |  |  |
| Sometimes/Rarely/Never | 518 | 6196 | 0.91 | 0.72-1.16 |
| Wore a facemask inside public spaces |  |  |  |  |
| Always/Mostly (ref) | 467 | 4356 |  |  |
| Sometimes/Rarely/Never | 165 | 2834 | 0.99 | 0.78-1.25 |

**Table S3: Adjusted* odds ratios and 95%-confidence intervals of risk factors for a positive SARS-CoV-2 test in fully vaccinated persons, comparison of symptomatic cases with asymptomatic controls in the Netherlands, 1 June 2021 – 28 February 2022. Adjusted for age group, gender, and week of testing.**

| Risk factor | Cases (n) | Controls (n) | OR | 95%-CI |
| --- | --- | --- | --- | --- |
| Total household size |  |  |  |  |
| 1 (ref) | 118 | 303 |  |  |
| 2-3 | 388 | 795 | 1.24 | 0.96-1.59 |
| 4+ | 166 | 239 | 1.71 | 1.25-2.33 |
| Living with children 0-12 |  |  |  |  |
| No (ref) | 582 | 1223 |  |  |
| Yes | 90 | 114 | 1.59 | 1.12-2.26 |
| Living with children 13-18 |  |  |  |  |
| No (ref) | 601 | 1188 |  |  |
| Yes | 71 | 149 | 1.04 | 0.75-1.43 |
| Healthcare worker |  |  |  |  |
| No (ref) | 517 | 1035 |  |  |
| Yes | 155 | 302 | 0.99 | 0.78-1.26 |
| Days went to work/study |  |  |  |  |
| Did not go to work/study (ref) | 115 | 220 |  |  |
| 1-5 days | 195 | 377 | 0.93 | 0.69-1.26 |
| 6-10 days | 166 | 358 | 0.80 | 0.59-1.10 |
| 10+ days | 35 | 73 | 0.82 | 0.50-1.33 |
| Does not have to go to work/study | 161 | 309 | 0.77 | 0.67-1.28 |
| Contacts at work/study |  |  |  |  |
| 0 contacts (ref) | 439 | 826 |  |  |
| 1-4 contacts | 86 | 204 | 0.79 | 0.59-1.05 |
| 5-9 contacts | 70 | 139 | 0.86 | 0.62-1.19 |
| 10-19 contacts | 36 | 79 | 0.76 | 0.50-1.18 |
| 20 or more contacts | 41 | 89 | 0.82 | 0.55-1.23 |
| Visited busy indoor locations |  |  |  |  |
| No (ref) | 378 | 821 |  |  |
| Yes | 294 | 516 | 1.15 | 0.94-1.41 |
| Visited busy outdoor locations |  |  |  |  |
| No (ref) | 548 | 1171 |  |  |
| Yes | 124 | 166 | 1.64 | 1.24-2.17 |
| Wore a facemask outside the house |  |  |  |  |
| Always/Mostly (ref) | 97 | 217 |  |  |
| Sometimes/Rarely/Never | 518 | 1024 | 1.16 | 0.88-1.53 |
| Wore a facemask everywhere inside |  |  |  |  |
| Always/Mostly (ref) | 467 | 962 |  |  |
| Sometimes/Rarely/Never | 165 | 329 | 1.09 | 0.83-1.44 |

**Figure S1: Adjusted* odds ratios and 95%-confidence intervals of risk factors for a positive SARS-CoV-2 test for persons who had at least one COVID-19 vaccine, comparison of symptomatic cases with symptomatic controls and asymptomatic controls in the Netherlands, 1 June 2021 – 28 February 2022. Adjusted for age group, gender, and week of testing.**


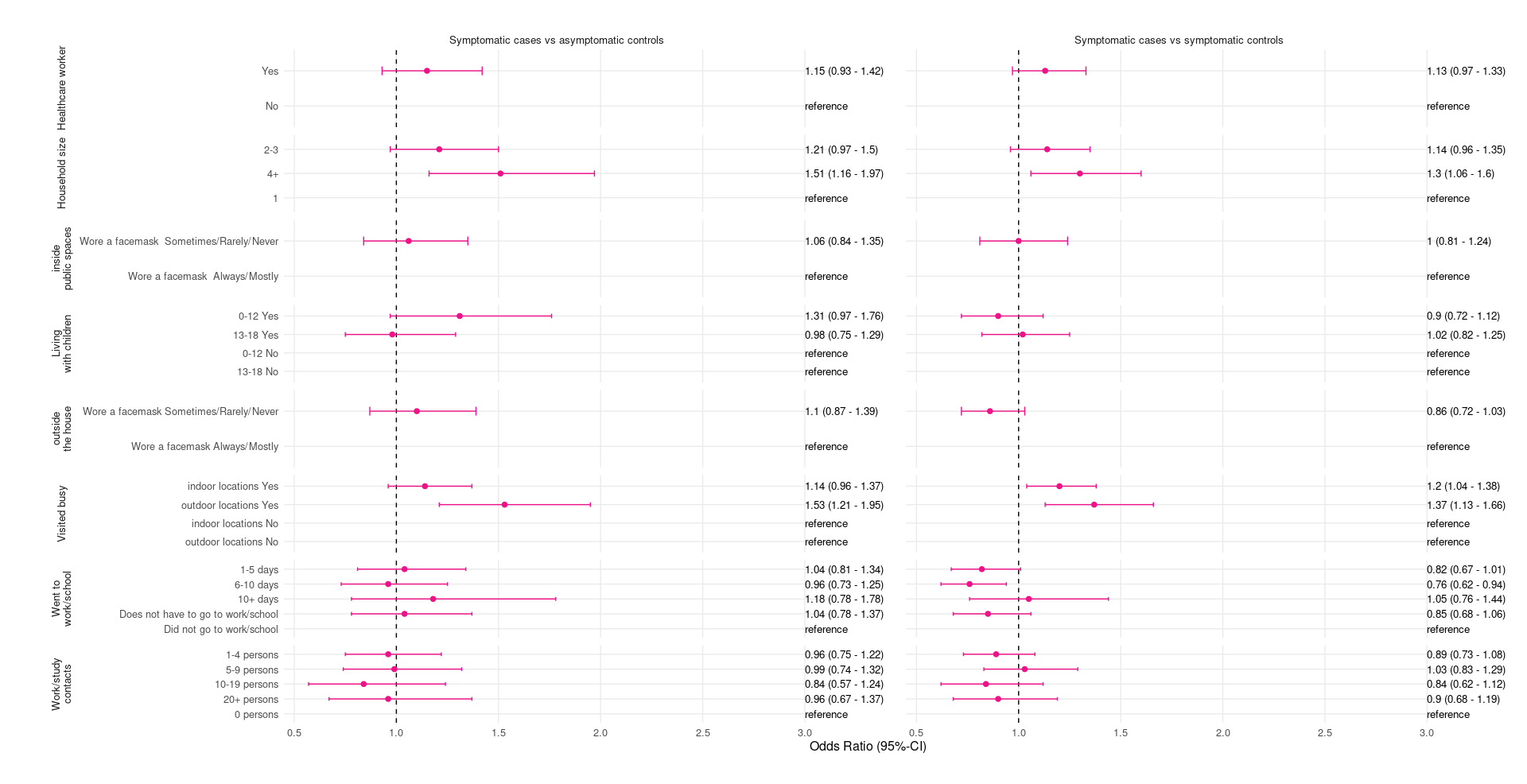


**Figure S2: Adjusted* odds ratios and 95%-confidence intervals of risk factors for a positive SARS-CoV-2 test in fully vaccinated persons, comparison of symptomatic cases with symptomatic controls and asymptomatic controls in the Netherlands, 1 June 2021 – 28 February 2022. Adjusted for age group, gender, days since vaccination, and week of testing.**


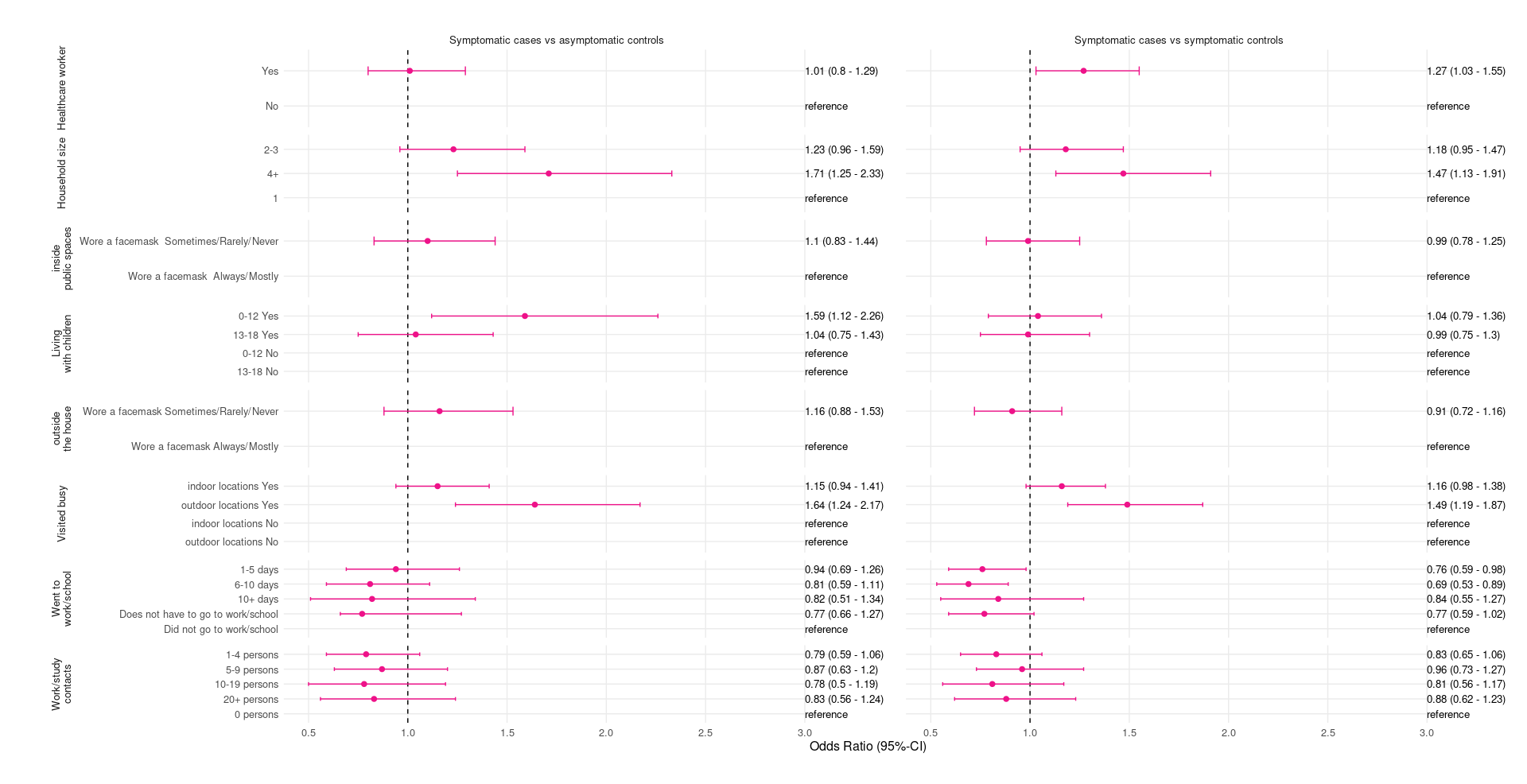


**Table S4: Adjusted* odds ratios and 95%-confidence intervals of risk factors for a positive SARS-CoV-2 test for persons who had at least one COVID-19 vaccine, comparison of symptomatic cases with symptomatic controls in the Netherlands, 1 June 2021 – 28 February 2022. Adjusted for age group, gender, and week of testing.**

| **Risk factor** | **Cases (n)** | **Controls (n)** | **OR** | **95%-CI** |
| --- | --- | --- | --- | --- |
| Total household size |  |  |  |  |
| 1 (ref) | 202 | 2064 |  |  |
| 2-3 | 641 | 5951 | 1.14 | 0.96-1.35 |
| 4+ | 251 | 2191 | 1.30 | 1.06-1.60 |
| Living with children 0-12 |  |  |  |  |
| No (ref) | 963 | 8726 |  |  |
| Yes | 131 | 1480 | 0.90 | 0.72-1.12 |
| Living with children 13-18 |  |  |  |  |
| No (ref) | 965 | 8928 |  |  |
| Yes | 129 | 1278 | 1.02 | 0.82-1.25 |
| Healthcare worker |  |  |  |  |
| No (ref) | 847 | 7989 |  |  |
| Yes | 247 | 2217 | 1.13 | 0.97-1.33 |
| Days went to work/study |  |  |  |  |
| Did not go to work/study (ref) | 185 | 1407 |  |  |
| 1-5 days | 320 | 3222 | 0.82 | 0.67-1.01 |
| 6-10 days | 277 | 2934 | 0.76 | 0.62-0.94 |
| 10+ days | 66 | 463 | 1.05 | 0.76-1.44 |
| Does not have to go to work/study | 246 | 2180 | 0.85 | 0.68-1.06 |
| Contacts at work/study |  |  |  |  |
| 0 contacts (ref) | 711 | 6276 |  |  |
| 1-4 contacts | 148 | 1552 | 0.89 | 0.73-1.08 |
| 5-9 contacts | 115 | 1034 | 1.03 | 0.83-1.29 |
| 10-19 contacts | 57 | 648 | 0.84 | 0.62-1.12 |
| 20 or more contacts | 63 | 696 | 0.90 | 0.68-1.19 |
| Visited busy indoor locations |  |  |  |  |
| No (ref) | 652 | 6187 |  |  |
| Yes | 442 | 4019 | 1.20 | 1.04-1.38 |
| Visited busy outdoor locations |  |  |  |  |
| No (ref) | 923 | 8720 |  |  |
| Yes | 171 | 1486 | 1.37 | 1.13-1.66 |
| Wore a facemask outside the house |  |  |  |  |
| Always/Mostly (ref) | 188 | 1185 |  |  |
| Sometimes/Rarely/Never | 819 | 8295 | 0.86 | 0.72-1.03 |
| Wore a facemask everywhere inside |  |  |  |  |
| Always/Mostly (ref) | 834 | 6383 |  |  |
| Sometimes/Rarely/Never | 211 | 3388 | 1.00 | 0.81-1.24 |

**Table S5: Adjusted* odds ratios and 95%-confidence intervals of risk factors for a positive SARS-CoV-2 test for persons who had at least one COVID-19 vaccine, comparison of symptomatic cases with asymptomatic controls in the Netherlands, 1 June 2021 – 28 February 2022. Adjusted for age group, gender, and week of testing.**

| **Risk factor** | **Cases (n)** | **Controls (n)** | **OR** | **95%-CI** |
| --- | --- | --- | --- | --- |
| Total household size |  |  |  |  |
| 1 (ref) | 202 | 406 |  |  |
| 2-3 | 641 | 1052 | 1.21 | 0.97-1.50 |
| 4+ | 251 | 354 | 1.51 | 1.16-1.97 |
| Living with children 0-12 |  |  |  |  |
| No (ref) | 963 | 1636 |  |  |
| Yes | 131 | 176 | 1.31 | 0.97-1.76 |
| Living with children 13-18 |  |  |  |  |
| No (ref) | 965 | 1595 |  |  |
| Yes | 129 | 217 | 0.98 | 0.75-1.29 |
| Healthcare worker |  |  |  |  |
| No (ref) | 847 | 1458 |  |  |
| Yes | 247 | 354 | 1.15 | 0.93-1.42 |
| Days went to work/study |  |  |  |  |
| Did not go to work/study (ref) | 185 | 306 |  |  |
| 1-5 days | 320 | 536 | 1.04 | 0.81-1.34 |
| 6-10 days | 277 | 477 | 0.96 | 0.73-1.25 |
| 10+ days | 66 | 94 | 1.18 | 0.78-1.78 |
| Does not have to go to work/study | 246 | 399 | 1.04 | -0.78-1.37 |
| Contacts at work/study |  |  |  |  |
| 0 contacts (ref) | 711 | 1165 |  |  |
| 1-4 contacts | 148 | 271 | 0.96 | 0.75-1.22 |
| 5-9 contacts | 115 | 169 | 0.99 | 0.74-1.32 |
| 10-19 contacts | 57 | 97 | 0.84 | 0.57-1.24 |
| 20 or more contacts | 63 | 110 | 0.96 | 0.67-1.37 |
| Visited busy indoor locations |  |  |  |  |
| No (ref) | 652 | 1139 |  |  |
| Yes | 442 | 673 | 1.14 | 0.96-1.37 |
| Visited busy outdoor locations |  |  |  |  |
| No (ref) | 923 | 1550 |  |  |
| Yes | 171 | 262 | 1.53 | 1.21-1.95 |
| Wore a facemask outside the house |  |  |  |  |
| Always/Mostly (ref) | 188 | 286 |  |  |
| Sometimes/Rarely/Never | 819 | 1407 | 1.10 | 0.87-1.39 |
| Wore a facemask everywhere inside |  |  |  |  |
| Always/Mostly (ref) | 834 | 1288 |  |  |
| Sometimes/Rarely/Never | 211 | 459 | 1.06 | 0.84-1.35 |

**Table S6: Adjusted* odds ratios and 95%-confidence intervals of risk factors for a positive SARS-CoV-2 test, comparison of symptomatic cases with symptomatic controls in the Netherlands, 1 June 2021 – 28 February 2022. Adjusted for age group, gender, days since vaccination, and week of testing.**

| **Risk factor** | **Cases (n)** | **Controls (n)** | **OR** | **95%-CI** |
| --- | --- | --- | --- | --- |
| Total household size |  |  |  |  |
| 1 (ref) | 118 | 1552 |  |  |
| 2-3 | 388 | 4389 | 1.18 | 0.95-1.47 |
| 4+ | 166 | 1620 | 1.47 | 1.13-1.91 |
| Living with children 0-12 |  |  |  |  |
| No (ref) | 582 | 6446 |  |  |
| Yes | 90 | 1085 | 1.04 | 0.79-1.36 |
| Living with children 13-18 |  |  |  |  |
| No (ref) | 601 | 6599 |  |  |
| Yes | 71 | 932 | 0.99 | 0.75-1.30 |
| Healthcare worker |  |  |  |  |
| No (ref) | 517 | 5898 |  |  |
| Yes | 155 | 1633 | 1.27 | 1.03-1.55 |
| Days went to work/study |  |  |  |  |
| Did not go to work/study (ref) | 115 | 951 |  |  |
| 1-5 days | 195 | 2394 | 0.76 | 0.59-0.98 |
| 6-10 days | 166 | 2221 | 0.69 | 0.53-0.89 |
| 10+ days | 35 | 348 | 0.84 | 0.55-1.27 |
| Does not have to go to work/study | 161 | 1617 | 0.77 | 0.59-1.02 |
| Contacts at work/study |  |  |  |  |
| 0 contacts (ref) | 439 | 4492 |  |  |
| 1-4 contacts | 86 | 1169 | 0.83 | 0.65-1.06 |
| 5-9 contacts | 70 | 816 | 0.96 | 0.73-1.27 |
| 10-19 contacts | 36 | 495 | 0.81 | 0.56-1.17 |
| 20 or more contacts | 41 | 559 | 0.88 | 0.62-1.23 |
| Visited busy indoor locations |  |  |  |  |
| No (ref) | 378 | 4299 |  |  |
| Yes | 294 | 3232 | 1.16 | 0.98-1.38 |
| Visited busy outdoor locations |  |  |  |  |
| No (ref) | 548 | 6438 |  |  |
| Yes | 124 | 1093 | 1.49 | 1.19-1.87 |
| Wore a facemask outside the house |  |  |  |  |
| Always/Mostly (ref) | 97 | 775 |  |  |
| Sometimes/Rarely/Never | 518 | 6196 | 0.91 | 0.72-1.16 |
| Wore a facemask everywhere inside |  |  |  |  |
| Always/Mostly (ref) | 467 | 4356 |  |  |
| Sometimes/Rarely/Never | 165 | 2834 | 0.99 | 0.78-1.25 |

**Table S7: Adjusted* odds ratios and 95%-confidence intervals of risk factors for a positive SARS-CoV-2 test, comparison of symptomatic cases with asymptomatic controls in the Netherlands, 1 June 2021 – 28 February 2022. Adjusted for age group, gender, days since vaccination, and week of testing.**

| **Risk factor** | **Cases (n)** | **Controls (n)** | **OR** | **95%-CI** |
| --- | --- | --- | --- | --- |
| Total household size |  |  |  |  |
| 1 (ref) | 118 | 303 |  |  |
| 2-3 | 388 | 795 | 1.23 | 0.96-1.59 |
| 4+ | 166 | 239 | 1.71 | 1.25-2.33 |
| Living with children 0-12 |  |  |  |  |
| No (ref) | 582 | 1223 |  |  |
| Yes | 90 | 114 | 1.59 | 1.12-2.26 |
| Living with children 13-18 |  |  |  |  |
| No (ref) | 601 | 1188 |  |  |
| Yes | 71 | 149 | 1.04 | 0.75-1.43 |
| Healthcare worker |  |  |  |  |
| No (ref) | 517 | 1035 |  |  |
| Yes | 155 | 302 | 1.01 | 0.80-1.29 |
| Days went to work/study |  |  |  |  |
| Did not go to work/study (ref) | 115 | 220 |  |  |
| 1-5 days | 195 | 377 | 0.94 | 0.69-1.26 |
| 6-10 days | 166 | 358 | 0.81 | 0.59-1.11 |
| 10+ days | 35 | 73 | 0.83 | 0.51-1.35 |
| Does not have to go to work/study | 161 | 309 | 0.77 | 0.66-1.27 |
| Contacts at work/study |  |  |  |  |
| 0 contacts (ref) | 439 | 826 |  |  |
| 1-4 contacts | 86 | 204 | 0.79 | 0.59-1.06 |
| 5-9 contacts | 70 | 139 | 0.87 | 0.63-1.20 |
| 10-19 contacts | 36 | 79 | 0.78 | 0.50-1.19 |
| 20 or more contacts | 41 | 89 | 0.83 | 0.56-1.24 |
| Visited busy indoor locations |  |  |  |  |
| No (ref) | 378 | 821 |  |  |
| Yes | 294 | 516 | 1.15 | 0.94-1.41 |
| Visited busy outdoor locations |  |  |  |  |
| No (ref) | 548 | 1171 |  |  |
| Yes | 124 | 166 | 1.64 | 1.24-2.17 |
| Wore a facemask outside the house |  |  |  |  |
| Always/Mostly (ref) | 97 | 217 |  |  |
| Sometimes/Rarely/Never | 518 | 1024 | 1.16 | 0.88-1.53 |
| Wore a facemask everywhere inside |  |  |  |  |
| Always/Mostly (ref) | 467 | 962 |  |  |
| Sometimes/Rarely/Never | 165 | 329 | 1.10 | 0.83-1.44 |

**Table S8: Adjusted* odds ratios and 95%-confidence intervals of risk factors for a positive SARS-CoV-2 test in fully vaccinated persons, comparison of symptomatic cases with symptomatic controls in the Netherlands, 1 June 2021 – 3 October 2021. Adjusted for age group, gender, and week of testing.**

| **Period 1**  **1-6-2021 until 3-10-2021** | | | | |
| --- | --- | --- | --- | --- |
| **Risk factor** | **Cases (n)** | **Controls (n)** | **OR** | **95%-CI** |
| Total household size |  |  |  |  |
| 1 (ref) | 26 | 434 |  |  |
| 2-3 | 92 | 1281 | 1.25 | 0.79-1.99 |
| 4+ | 61 | 465 | 1.60 | 0.97-2.66 |
| Living with children 0-12 |  |  |  |  |
| No (ref) | 160 | 1894 |  |  |
| Yes | 19 | 286 | 0.94 | 0.54-1.65 |
| Living with children 13-18 |  |  |  |  |
| No (ref) | 153 | 1923 |  |  |
| Yes | 26 | 257 | 1.27 | 0.79-2.04 |
| Healthcare worker |  |  |  |  |
| No (ref) | 132 | 1672 |  |  |
| Yes | 47 | 508 | 1.05 | 0.72-1.52 |
| Days went to work/study |  |  |  |  |
| Did not go to work/study (ref) | 33 | 332 |  |  |
| 1-5 days | 50 | 611 | 0.74 | 0.46-1.20 |
| 6-10 days | 37 | 585 | 0.55 | 0.33-0.92 |
| 10+ days | 12 | 74 | 1.30 | 0.61-2.77 |
| Does not have to go to work/study | 47 | 578 | 0.75 | 0.45-1.26 |
| Contacts at work/study |  |  |  |  |
| 0 contacts (ref) | 123 | 1499 |  |  |
| 1-4 contacts | 23 | 279 | 0.90 | 0.55-1.47 |
| 5-9 contacts | 16 | 169 | 1.10 | 0.62-1.97 |
| 10-19 contacts | 8 | 112 | 0.73 | 0.34-1.58 |
| 20 or more contacts | 9 | 121 | 0.91 | 0.44-1.90 |
| Visited busy indoor locations |  |  |  |  |
| No (ref) | 111 | 1647 |  |  |
| Yes | 68 | 533 | 1.58 | 1.10-2.26 |
| Visited busy outdoor locations |  |  |  |  |
| No (ref) | 99 | 1589 |  |  |
| Yes | 80 | 591 | 1.58 | 1.14-2.19 |
| Wore a facemask outside the house |  |  |  |  |
| Always/Mostly (ref) | 15 | 177 |  |  |
| Sometimes/Rarely/Never | 152 | 1816 | 1.03 | 0.58-1.83 |
| Wore a facemask everywhere inside |  |  |  |  |
| Always/Mostly (ref) | 87 | 1013 |  |  |
| Sometimes/Rarely/Never | 76 | 1023 | 0.98 | 0.69-1.40 |

**Table S9: Adjusted* odds ratios and 95%-confidence intervals of risk factors for a positive SARS-CoV-2 test in fully vaccinated persons, comparison of symptomatic cases with symptomatic controls in the Netherlands, 4 October 2021 – 2 January 2022. Adjusted for age group, gender, and week of testing.**

| **Period 2**  **4-10-2021 until 2-1-2022** | | | | |
| --- | --- | --- | --- | --- |
| **Risk factor** | **Cases (n)** | **Controls (n)** | **OR** | **95%-CI** |
| Total household size |  |  |  |  |
| 1 (ref) | 80 | 1017 |  |  |
| 2-3 | 247 | 2949 | 1.06 | 0.81-1.38 |
| 4+ | 68 | 1099 | 1.03 | 0.73-1.47 |
| Living with children 0-12 |  |  |  |  |
| No (ref) | 347 | 4304 |  |  |
| Yes | 48 | 761 | 0.93 | 0.64-1.33 |
| Living with children 13-18 |  |  |  |  |
| No (ref) | 366 | 4422 |  |  |
| Yes | 29 | 643 | 0.68 | 0.45-1.02 |
| Healthcare worker |  |  |  |  |
| No (ref) | 301 | 3986 |  |  |
| Yes | 94 | 1079 | 1.45 | 1.12-1.87 |
| Days went to work/study |  |  |  |  |
| Did not go to work/study (ref) | 52 | 545 |  |  |
| 1-5 days | 122 | 1713 | 0.90 | 0.63-1.27 |
| 6-10 days | 109 | 1549 | 0.98 | 0.68-1.40 |
| 10+ days | 18 | 260 | 0.95 | 0.54-1.68 |
| Does not have to go to work/study | 94 | 103 | 0.80 | 0.55-1.16 |
| Contacts at work/study |  |  |  |  |
| 0 contacts (ref) | 242 | 2810 |  |  |
| 1-4 contacts | 59 | 851 | 0.99 | 0.73-1.35 |
| 5-9 contacts | 45 | 616 | 1.12 | 0.80-1.58 |
| 10-19 contacts | 21 | 368 | 0.89 | 0.55-1.42 |
| 20 or more contacts | 28 | 420 | 1.02 | 0.67-1.54 |
| Visited busy indoor locations |  |  |  |  |
| No (ref) | 211 | 2484 |  |  |
| Yes | 184 | 2581 | 0.98 | 0.79-1.21 |
| Visited busy outdoor locations |  |  |  |  |
| No (ref) | 366 | 4580 |  |  |
| Yes | 29 | 485 | 0.96 | 0.65-1.43 |
| Wore a facemask outside the house |  |  |  |  |
| Always/Mostly (ref) | 57 | 539 |  |  |
| Sometimes/Rarely/Never | 301 | 4164 | 0.88 | 0.64-1.19 |
| Wore a facemask everywhere inside |  |  |  |  |
| Always/Mostly (ref) | 287 | 3065 |  |  |
| Sometimes/Rarely/Never | 85 | 1806 | 0.84 | 0.60-1.16 |

**Table S10: Adjusted* odds ratios and 95%-confidence intervals of risk factors for a positive SARS-CoV-2 test in fully vaccinated persons, comparison of symptomatic cases with asymptomatic controls in the Netherlands, 1 June 2021 – 3 October 2021. Adjusted for age group, gender, and week of testing.**

| **Period 1**  **1-6-2021 until 3-10-2021** | | | | |
| --- | --- | --- | --- | --- |
| **Risk factor** | **Cases (n)** | **Controls (n)** | **OR** | **95%-CI** |
| Total household size |  |  |  |  |
| 1 (ref) | 26 | 77 |  |  |
| 2-3 | 92 | 223 | 1.32 | 0.77-2.26 |
| 4+ | 61 | 65 | 2.13 | 1.17-3.89 |
| Living with children 0-12 |  |  |  |  |
| No (ref) | 160 | 343 |  |  |
| Yes | 19 | 22 | 2.05 | 0.98-4.28 |
| Living with children 13-18 |  |  |  |  |
| No (ref) | 153 | 322 |  |  |
| Yes | 26 | 43 | 1.36 | 0.77-2.42 |
| Healthcare worker |  |  |  |  |
| No (ref) | 132 | 271 |  |  |
| Yes | 47 | 94 | 0.89 | 0.57-1.40 |
| Days went to work/study |  |  |  |  |
| Did not go to work/study (ref) | 33 | 69 |  |  |
| 1-5 days | 50 | 100 | 0.91 | 0.51-1.62 |
| 6-10 days | 37 | 84 | 0.74 | 0.40-1.37 |
| 10+ days | 12 | 20 | 0.75 | 0.31-1.82 |
| Does not have to go to work/study | 47 | 92 | 1.13 | 0.60-2.10 |
| Contacts at work/study |  |  |  |  |
| 0 contacts (ref) | 123 | 246 |  |  |
| 1-4 contacts | 23 | 46 | 0.84 | 0.47-1.52 |
| 5-9 contacts | 16 | 31 | 0.71 | 0.36-1.39 |
| 10-19 contacts | 8 | 18 | 0.70 | 0.28-1.75 |
| 20 or more contacts | 9 | 24 | 0.57 | 0.25-1.33 |
| Visited busy indoor locations |  |  |  |  |
| No (ref) | 111 | 271 |  |  |
| Yes | 68 | 94 | 1.25 | 0.81-1.93 |
| Visited busy outdoor locations |  |  |  |  |
| No (ref) | 99 | 262 |  |  |
| Yes | 80 | 103 | 1.67 | 1.12-2.48 |
| Wore a facemask outside the house |  |  |  |  |
| Always/Mostly (ref) | 15 | 55 |  |  |
| Sometimes/Rarely/Never | 152 | 282 | 1.50 | 0.79-2.84 |
| Wore a facemask everywhere inside |  |  |  |  |
| Always/Mostly (ref) | 87 | 210 |  |  |
| Sometimes/Rarely/Never | 76 | 138 | 1.11 | 0.73-1.68 |

**Table S11: Adjusted* odds ratios and 95%-confidence intervals of risk factors for a positive SARS-CoV-2 test in fully vaccinated persons, comparison of symptomatic cases with asymptomatic controls in the Netherlands, 4 October 2021 – 2 January 2022. Adjusted for age group, gender, and week of testing.**

| **Period 2**  **4-10-2021 until 2-1-2022** | | | | |
| --- | --- | --- | --- | --- |
| **Risk factor** | **Cases (n)** | **Controls (n)** | **OR** | **95%-CI** |
| Total household size |  |  |  |  |
| 1 (ref) | 80 | 202 |  |  |
| 2-3 | 247 | 506 | 1.19 | 0.87-1.61 |
| 4+ | 68 | 145 | 1.33 | 0.88-2.01 |
| Living with children 0-12 |  |  |  |  |
| No (ref) | 347 | 772 |  |  |
| Yes | 48 | 81 | 1.33 | 0.85-2.09 |
| Living with children 13-18 |  |  |  |  |
| No (ref) | 366 | 765 |  |  |
| Yes | 29 | 88 | 0.83 | 0.52-1.32 |
| Healthcare worker |  |  |  |  |
| No (ref) | 301 | 658 |  |  |
| Yes | 94 | 195 | 1.03 | 0.76-1.39 |
| Days went to work/study |  |  |  |  |
| Did not go to work/study (ref) | 52 | 112 |  |  |
| 1-5 days | 122 | 240 | 1.11 | 0.74-1.67 |
| 6-10 days | 109 | 253 | 0.95 | 0.62-1.44 |
| 10+ days | 18 | 50 | 0.89 | 0.46-1.72 |
| Does not have to go to work/study | 94 | 198 | 0.86 | 0.56-1.34 |
| Contacts at work/study |  |  |  |  |
| 0 contacts (ref) | 242 | 501 |  |  |
| 1-4 contacts | 59 | 139 | 0.94 | 0.65-1.34 |
| 5-9 contacts | 45 | 98 | 0.97 | 0.65-1.45 |
| 10-19 contacts | 21 | 56 | 0.81 | 0.47-1.39 |
| 20 or more contacts | 28 | 59 | 1.00 | 0.61-1.65 |
| Visited busy indoor locations |  |  |  |  |
| No (ref) | 211 | 481 |  |  |
| Yes | 184 | 372 | 1.06 | 0.82-1.36 |
| Visited busy outdoor locations |  |  |  |  |
| No (ref) | 366 | 800 |  |  |
| Yes | 29 | 53 | 1.16 | 0.72-1.88 |
| Wore a facemask outside the house |  |  |  |  |
| Always/Mostly (ref) | 57 | 133 |  |  |
| Sometimes/Rarely/Never | 301 | 659 | 1.03 | 0.72-1.47 |
| Wore a facemask everywhere inside |  |  |  |  |
| Always/Mostly (ref) | 287 | 640 |  |  |
| Sometimes/Rarely/Never | 85 | 187 | 0.89 | 0.60-1.32 |

**Table S12: Adjusted* odds ratios and 95%-confidence intervals of risk factors for a positive SARS-CoV-2 test in fully vaccinated persons, comparison of symptomatic cases with symptomatic controls in the Netherlands, 28 June 2021 – 2 August 2021. Adjusted for age group, gender, and week of testing.**

| **summerwave**  **28-6-2021 until 2-8-2021** | | | | |
| --- | --- | --- | --- | --- |
| **Risk factor** | **Cases (n)** | **Controls (n)** | **OR** | **95%-CI** |
| Total household size |  |  |  |  |
| 1 (ref) | 16 | 146 |  |  |
| 2-3 | 54 | 454 | 1.18 | 0.63-2.19 |
| 4+ | 39 | 156 | 1.47 | 0.76-2.85 |
| Living with children 0-12 |  |  |  |  |
| No (ref) | 99 | 666 |  |  |
| Yes | 10 | 90 | 1.01 | 0.46-2.25 |
| Living with children 13-18 |  |  |  |  |
| No (ref) | 96 | 680 |  |  |
| Yes | 13 | 76 | 0.97 | 0.49-1.91 |
| Healthcare worker |  |  |  |  |
| No (ref) | 82 | 552 |  |  |
| Yes | 27 | 204 | 0.89 | 0.54-1.47 |
| Days went to work/study |  |  |  |  |
| Did not go to work/study (ref) | 13 | 97 |  |  |
| 1-5 days | 32 | 219 | 0.89 | 0.43-1.84 |
| 6-10 days | 21 | 197 | 0.51 | 0.24-1.11 |
| 10+ days | 9 | 25 | 1.58 | 0.57-4.36 |
| Does not have to go to work/study | 34 | 218 | 1.15 | 0.54-2.45 |
| Contacts at work/study |  |  |  |  |
| 0 contacts (ref) | 78 | 509 |  |  |
| 1-4 contacts | 13 | 112 | 0.59 | 0.31-1.13 |
| 5-9 contacts | 9 | 55 | 0.82 | 0.37-1.81 |
| 10-19 contacts | 5 | 41 | 0.62 | 0.23-1.69 |
| 20 or more contacts | 4 | 39 | 0.53 | 0.18-1.60 |
| Visited busy indoor locations |  |  |  |  |
| No (ref) | 59 | 608 |  |  |
| Yes | 50 | 148 | 1.79 | 1.11-2.87 |
| Visited busy outdoor locations |  |  |  |  |
| No (ref) | 56 | 538 |  |  |
| Yes | 53 | 218 | 1.81 | 1.17-2.81 |
| Wore a facemask outside the house |  |  |  |  |
| Always/Mostly (ref) | 11 | 98 |  |  |
| Sometimes/Rarely/Never | 91 | 589 | 0.86 | 0.42-1.75 |
| Wore a facemask everywhere inside |  |  |  |  |
| Always/Mostly (ref) | 53 | 416 |  |  |
| Sometimes/Rarely/Never | 46 | 282 | 1.16 | 0.73-1.84 |

**Table S13: Adjusted* odds ratios and 95%-confidence intervals of risk factors for a positive SARS-CoV-2 test in fully vaccinated persons, comparison of symptomatic cases with symptomatic controls in the Netherlands, 4 October 2021 – 20 December 2021. Adjusted for age group, gender, and week of testing.**

| **fallwave**  **4-10-2021 until 20-12-2021** | | | | |
| --- | --- | --- | --- | --- |
| **Risk factor** | **Cases (n)** | **Controls (n)** | **OR** | **95%-CI** |
| Total household size |  |  |  |  |
| 1 (ref) | 76 | 968 |  |  |
| 2-3 | 236 | 2846 | 1.05 | 0.80-1.38 |
| 4+ | 67 | 1049 | 1.08 | 0.76-1.55 |
| Living with children 0-12 |  |  |  |  |
| No (ref) | 332 | 4137 |  |  |
| Yes | 47 | 726 | 0.99 | 0.68-1.43 |
| Living with children 13-18 |  |  |  |  |
| No (ref) | 350 | 4253 |  |  |
| Yes | 29 | 610 | 0.72 | 0.47-1.08 |
| Healthcare worker |  |  |  |  |
| No (ref) | 285 | 3809 |  |  |
| Yes | 94 | 1054 | 1.52 | 1.17-1.97 |
| Days went to work/study |  |  |  |  |
| Did not go to work/study (ref) | 48 | 500 |  |  |
| 1-5 days | 117 | 1640 | 0.91 | 0.64-1.30 |
| 6-10 days | 104 | 1506 | 0.97 | 0.67-1.41 |
| 10+ days | 18 | 249 | 1.00 | 0.56-1.78 |
| Does not have to go to work/study | 92 | 968 | 0.81 | 0.55-1.18 |
| Contacts at work/study |  |  |  |  |
| 0 contacts (ref) | 231 | 2676 |  |  |
| 1-4 contacts | 55 | 815 | 0.97 | 0.71-1.33 |
| 5-9 contacts | 45 | 597 | 1.17 | 0.83-1.65 |
| 10-19 contacts | 21 | 361 | 0.91 | 0.57-1.46 |
| 20 or more contacts | 27 | 414 | 1.00 | 0.66-1.53 |
| Visited busy indoor locations |  |  |  |  |
| No (ref) | 202 | 2355 |  |  |
| Yes | 177 | 2508 | 0.97 | 0.78-1.20 |
| Visited busy outdoor locations |  |  |  |  |
| No (ref) | 351 | 4390 |  |  |
| Yes | 28 | 473 | 0.96 | 0.64-1.43 |
| Wore a facemask outside the house |  |  |  |  |
| Always/Mostly (ref) | 57 | 509 |  |  |
| Sometimes/Rarely/Never | 287 | 4008 | 0.83 | 0.61-1.14 |
| Wore a facemask everywhere inside |  |  |  |  |
| Always/Mostly (ref) | 271 | 2865 |  |  |
| Sometimes/Rarely/Never | 85 | 1805 | 0.84 | 0.61-1.17 |

**Table S14: Adjusted* odds ratios and 95%-confidence intervals of risk factors for a positive SARS-CoV-2 test in fully vaccinated persons, comparison of symptomatic cases with asymptomatic controls in the Netherlands, 28 June 2021 – 2 August 2021. Adjusted for age group, gender, and week of testing.**

| **summerwave**  **28-6-2021 until 2-8-2021** | | | | |
| --- | --- | --- | --- | --- |
| **Risk factor** | **Cases (n)** | **Controls (n)** | **OR** | **95%-CI** |
| Total household size |  |  |  |  |
| 1 (ref) | 16 | 42 |  |  |
| 2-3 | 54 | 115 | 1.27 | 0.63-2.56 |
| 4+ | 39 | 43 | 1.74 | 0.81-3.73 |
| Living with children 0-12 |  |  |  |  |
| No (ref) | 99 | 189 |  |  |
| Yes | 10 | 11 | 2.61 | 0.93-7.37 |
| Living with children 13-18 |  |  |  |  |
| No (ref) | 96 | 176 |  |  |
| Yes | 13 | 24 | 1.11 | 0.50-2.45 |
| Healthcare worker |  |  |  |  |
| No (ref) | 82 | 148 |  |  |
| Yes | 27 | 52 | 1.02 | 0.55-1.88 |
| Days went to work/study |  |  |  |  |
| Did not go to work/study (ref) | 13 | 33 |  |  |
| 1-5 days | 32 | 52 | 1.24 | 0.54-2.87 |
| 6-10 days | 21 | 47 | 0.83 | 0.35-2.01 |
| 10+ days | 9 | 15 | 0.85 | 0.28-2.60 |
| Does not have to go to work/study | 34 | 53 | 1.43 | 0.59-3.44 |
| Contacts at work/study |  |  |  |  |
| 0 contacts (ref) | 78 | 134 |  |  |
| 1-4 contacts | 13 | 27 | 0.69 | 0.32-1.49 |
| 5-9 contacts | 9 | 18 | 0.60 | 0.25-1.48 |
| 10-19 contacts | 5 | 7 | 0.84 | 0.24-2.91 |
| 20 or more contacts | 4 | 14 | 0.37 | 0.11-1.20 |
| Visited busy indoor locations |  |  |  |  |
| No (ref) | 59 | 140 |  |  |
| Yes | 50 | 60 | 1.23 | 0.70-2.15 |
| Visited busy outdoor locations |  |  |  |  |
| No (ref) | 56 | 139 |  |  |
| Yes | 53 | 61 | 1.85 | 1.10-3.10 |
| Wore a facemask outside the house |  |  |  |  |
| Always/Mostly (ref) | 11 | 21 |  |  |
| Sometimes/Rarely/Never | 91 | 164 | 0.82 | 0.35-1.91 |
| Wore a facemask everywhere inside |  |  |  |  |
| Always/Mostly (ref) | 53 | 113 |  |  |
| Sometimes/Rarely/Never | 46 | 78 | 1.14 | 0.67-1.96 |

**Table S15: Adjusted* odds ratios and 95%-confidence intervals of risk factors for a positive SARS-CoV-2 test in fully vaccinated persons, comparison of symptomatic cases with asymptomatic controls in the Netherlands, 4 October 2021 – 20 December 2021. Adjusted for age group, gender, and week of testing.**

| **fallwave**  **4-10-2021 until 20-12-2021** | | | | |
| --- | --- | --- | --- | --- |
| **Risk factor** | **Cases (n)** | **Controls (n)** | **OR** | **95%-CI** |
| Total household size |  |  |  |  |
| 1 (ref) | 76 | 179 |  |  |
| 2-3 | 236 | 465 | 1.16 | 0.85-1.59 |
| 4+ | 67 | 132 | 1.35 | 0.89-2.07 |
| Living with children 0-12 |  |  |  |  |
| No (ref) | 332 | 702 |  |  |
| Yes | 47 | 74 | 1.40 | 0.88-2.23 |
| Living with children 13-18 |  |  |  |  |
| No (ref) | 350 | 699 |  |  |
| Yes | 29 | 77 | 0.88 | 0.55-1.41 |
| Healthcare worker |  |  |  |  |
| No (ref) | 285 | 592 |  |  |
| Yes | 94 | 184 | 1.08 | 0.79-1.46 |
| Days went to work/study |  |  |  |  |
| Did not go to work/study (ref) | 48 | 96 |  |  |
| 1-5 days | 117 | 211 | 1.14 | 0.74-1.74 |
| 6-10 days | 104 | 232 | 0.94 | 0.61-1.46 |
| 10+ days | 18 | 48 | 0.91 | 0.47-1.78 |
| Does not have to go to work/study | 92 | 189 | 0.87 | 0.55-1.36 |
| Contacts at work/study |  |  |  |  |
| 0 contacts (ref) | 231 | 450 |  |  |
| 1-4 contacts | 55 | 121 | 0.92 | 0.64-1.34 |
| 5-9 contacts | 45 | 95 | 0.99 | 0.66-1.48 |
| 10-19 contacts | 21 | 55 | 0.81 | 0.47-1.40 |
| 20 or more contacts | 27 | 55 | 1.00 | 0.60-1.65 |
| Visited busy indoor locations |  |  |  |  |
| No (ref) | 202 | 429 |  |  |
| Yes | 177 | 347 | 1.03 | 0.79-1.33 |
| Visited busy outdoor locations |  |  |  |  |
| No (ref) | 351 | 725 |  |  |
| Yes | 28 | 51 | 1.13 | 0.70-1.84 |
| Wore a facemask outside the house |  |  |  |  |
| Always/Mostly (ref) | 57 | 115 |  |  |
| Sometimes/Rarely/Never | 287 | 606 | 1.36 | 0.65-1.36 |
| Wore a facemask everywhere inside |  |  |  |  |
| Always/Mostly (ref) | 271 | 563 |  |  |
| Sometimes/Rarely/Never | 85 | 187 | 0.91 | 0.61-1.35 |
